# Co-Infection by Chytrid Fungus and *Ranaviruses* in Wild and Harvested Frogs in the Tropical Andes

**DOI:** 10.1371/journal.pone.0145864

**Published:** 2016-01-04

**Authors:** Robin W. Warne, Brandon LaBumbard, Seth LaGrange, Vance T. Vredenburg, Alessandro Catenazzi

**Affiliations:** 1 Southern Illinois University, Department of Zoology, 1125 Lincoln Dr., MC6501, Carbondale, IL, 62901, United States of America; 2 Department of Biology, San Francisco State University, San Francisco, CA, 94132, United States of America; University of South Dakota, UNITED STATES

## Abstract

While global amphibian declines are associated with the spread of *Batrachochytrium dendrobatidis* (Bd), undetected concurrent co-infection by other pathogens may be little recognized threats to amphibians. Emerging viruses in the genus *Ranavirus* (Rv) also cause die-offs of amphibians and other ectotherms, but the extent of their distribution globally, or how co-infections with Bd impact amphibians are poorly understood. We provide the first report of Bd and Rv co-infection in South America, and the first report of Rv infections in the amphibian biodiversity hotspot of the Peruvian Andes, where Bd is associated with extinctions. Using these data, we tested the hypothesis that Bd or Rv parasites facilitate co-infection, as assessed by parasite abundance or infection intensity within individual adult frogs. Co-infection occurred in 30% of stream-dwelling frogs; 65% were infected by Bd and 40% by Rv. Among terrestrial, direct-developing *Pristimantis* frogs 40% were infected by Bd, 35% by Rv, and 20% co-infected. In *Telmatobius* frogs harvested for the live-trade 49% were co-infected, 92% were infected by Bd, and 53% by Rv. Median Bd and Rv loads were similar in both wild (Bd = 10^1.2^ Ze, Rv = 10^2.3^ viral copies) and harvested frogs (Bd = 10^3.1^ Ze, Rv = 10^2.7^ viral copies). While neither parasite abundance nor infection intensity were associated with co-infection patterns in adults, these data did not include the most susceptible larval and metamorphic life stages. These findings suggest Rv distribution is global and that co-infection among these parasites may be common. These results raise conservation concerns, but greater testing is necessary to determine if parasite interactions increase amphibian vulnerability to secondary infections across differing life stages, and constitute a previously undetected threat to declining populations. Greater surveillance of parasite interactions may increase our capacity to contain and mitigate the impacts of these and other wildlife diseases.

## Introduction

Emerging infectious diseases are threatening biodiversity [1]. In particular, the recent emergence of the chytrid fungus *Batrachochytrium dendrobatidis* (Bd) is linked to extirpations and even extinctions of amphibians globally; especially in Central America [2, 3], the tropical Andes [4, 5], the western US [6] and Australia [7]. In addition, emerging viral pathogens in the genus *Ranavirus* (Rv) have caused massive die-offs of amphibians and other ectothermic vertebrates in North America and Europe [8–10], with fewer reports in other regions [11–14]. More recently, Bd and Rv have been reported to co-occur in varied habitats [15, 16], and to co-infect individual hosts [13]. Beyond these reports, however, little is known about the prevalence of *ranaviruses* in other regions of high amphibian biodiversity, the extent to which Bd and Rv co-infect amphibians, modes of disease spread, or the threat that any such co-infections present for amphibians.

The tropical Andes in South America are among the most species rich regions for amphibians on Earth. Chytridiomycosis has been associated with population declines of frogs throughout the Andes [4, 17], and Bd has been affecting frogs in Peru since at least 1999 [5]. Bd is widely distributed across elevation gradients and ecosystems in the Andes despite variation in precipitation and thermal regimes that can influence the growth of Bd.

The live-trade of comestible amphibians in the Andes of Peru and Bolivia may be a critical vector for the spread of Bd. In particular, we recently found that threatened frogs *Telmatobius marmoratus* that are harvested for human consumption [18–20] were heavily infected by Bd [21]. Because these wild-caught animals are kept in high-density conditions that increase Bd transmission, and they are transported across regional markets, the live-trade is a probable route for disease spillover and spread to wild amphibian populations. If Rv exhibits similar prevalence patterns to Bd or if it is similarly associated with the live-trade in Peru is unknown. However, disease spillover from confined market to wild amphibian populations is a probable route for the invasion of *ranaviruses* in the United States [22].

*Ranavirus* outbreaks are thought to occur globally and can be highly virulent, resulting in mass-mortality events that affect multiple species of ectothermic vertebrates [23–25]. In addition, *ranaviruses* are not host-specific, so a single strain can infect fish, reptiles and amphibians [23, 26–29]. Therefore, Rv can threaten entire wetland, stream and riparian communities, as was reported for a recent outbreak in Spain [10]. Rv disease outbreaks are likely influenced by variation in susceptibility among species [30] and life stages [31, 32]. Environmental stressors like altered habitats, pollution and climate change are also thought to contribute to Rv outbreaks [9, 33]. In addition, another but little explored factor that may also contribute to the emergence and spread of diseases such as Rv is prior or concurrent infection by other parasites like Bd.

Interactions among one or more co-infecting parasites can be antagonistic or facilitative, by which they may influence disease related morbidity and mortality in their hosts [34]. Antagonistic interactions may occur through resource competition or induction of cross-effective immune responses within the host [35, 36]. Facilitative interactions in contrast, may increase infection and disease in a host via immunosuppression and resource depletion, thereby increasing infectious spread of one or both parasites [34–37]. Indeed, co-infection by multiple parasites is likely common for most wild animals [38–40] and has been associated with increased susceptibility to subsequent infections and increases in disease-related mortality rates in mammals [35, 37, 41]. There are good reasons to suspect that both Rv and Bd could also facilitate co-infections among one another, as well as other parasites in amphibians [13, 15]. Amphibian immunity to Rv and Bd include both innate and adaptive effectors. For example, while T-cell and antibody mediated immunity are critical to fighting Rv infection [42, 43], Bd can suppress these immune factors because it inhibits lymphocyte production and induces apoptosis in these adaptive immune cells [44]. Conversely, innate immunity that includes phagocytic cells, lysozymes, and antimicrobial peptides are central to amphibian immunity against chytridiomycosis. However, Rv evades the amphibian immune system by targeting and infecting macrophages in addition to system wide disruption of amphibian tissues [45]. These direct impacts on immune function suggest that both Bd and Rv could facilitate co-infection via immunosuppression, as well as through general host tissue disruption and resource depletion. To shed light on these little explored dynamics, we examined patterns of co-infection between Bd and Rv in wild frogs and in frogs harvested for the live trade in Peru. Using these data, we tested the hypothesis that Bd or Rv parasite abundance within individuals contributes to the likelihood of co-infection among adult frogs. We also tested for the potential of co-infection to increase the infection intensity of both parasites, which could suggest facilitation.

## Methods

### Animal sampling

Our sampling design encompassed two distinct study systems: wild-harvested live frogs from the San Pedro city market in Cusco [21], and wild frogs in montane forests of the eastern slopes of the Andes at the Kosñipata Valley near Manu National Park [17]. The frogs used in this study were previously obtained as part of broader efforts to document Bd susceptibility patterns in wild and captive trade amphibians; this work thus maximizes the use of these specimens. All wild and captive frog collections and sampling were conducted under the approval of the Southern Illinois University Animal Care and Use Committee (IACUC) and the Peruvian Ministry of Agriculture (permit #292-2014-MINAGRI-DGFFS/DGEFFS). All frogs obtained from the city market were individuals of *Telmatobius marmoratus* (n = 87), a Vulnerable species according to the IUCN Red List [46]. These *T*. *marmoratus* frogs were obtained alive from 22 June 2012 to 23 July 2013, and then euthanized by a 20% benzocaine overdose following guidelines of the Herpetological Animal Care and Use Committee [47]. Although the exact origin of these frogs is unknown, it is likely to be within the region of Cusco, because the species is common throughout this region and inhabits streams surrounding the city. The species naturally occurs in creeks, streams, ponds and wetlands, predominantly in high-elevation grasslands and other open areas.

The second study region is located on the eastern slopes of the Cordillera de Paucartambo, Cusco in the drainage basin of the Río Kosñipata, southern Peru [17]. The study sites are located in the Kosñipata valley and range from the submontane forest in the Amazonian foothill of the Andes at 945 m to the cloud forest at 2410 m [17]. We hand-captured 94 frogs from 12 June to 31 July 2013 during nocturnal surveys conducted along the Paucartambo-Shintuya road (S1 Fig). The road lies at the southern border of Manu National Park and of its buffer zone. Manu NP covers 17,163 km^2^ of Amazonian lowland, montane and high-elevation Andean habitats between 300 m and 4020 m elevation, and is the protected area harboring the largest number of amphibians on Earth [48]. All frogs were first swabbed for Bd following standard methods (see below) and then euthanized by a 20% benzocaine overdose following guidelines of the Herpetological Animal Care and Use Committee [47] for tissue collection needed for Rv analysis (see below).

### Bd qPCR

We collected skin swabs [49] by stroking a dry synthetic cotton swab across the skin of each frog; a standard technique used in previous surveys [17]. The swabbing protocol included 5 strokes on each side of the abdominal midline, 5 strokes on the inner thighs of each hind leg, and 5 strokes on the foot webbing of each hind leg for a total of 30 strokes/frog. We extracted DNA from swabs by using Prepman Ultra® (Life Technologies), and analyzed extracts with a real-time PCR (qPCR) assay on a StepOnePlus™ Real-Time PCR System (Life Technologies) to quantify the amount of genomic material [50].The assay uses genetic markers specific for Bd (primers of ITS gene); and compares each sample to a set of standards (four serial dilutions at concentrations from100 to 0.1 zoospore genomic equivalents, each in triplicate) to calculate a genomic equivalent. Each plate also includes four negative controls. To calculate Bd infection intensity, we multiplied the qPCR score by 80 to account for subsampling and dilution that occurred during the DNA extraction, resulting in a zoospore equivalent (Z_e_) estimate for each frog (Briggs et al., 2010; Vredenburg et al., 2010).

### *Ranavirus* qPCR

qPCR was used to detect and quantify the presence of *ranavirus* DNA in the livers of frogs [32, 51]. DNA was extracted and purified from the livers, stored in ethanol, by DNeasy® Blood and Tissue mini-spin extraction kits (Qiagen Inc.), following the manufacturers protocol. To standardize DNA used in qPCR analysis, total purified DNA was quantified with a Take3™ Microvolume Plate on an Epoch Spectrophotometer (BioTek Instruments INC) and diluted to 20 ng DNA · μL^-1^. Samples were assayed on a StepOnePlus in duplicate using TaqMan primers and probes that amplify a 70-bp region within the *ranavirus* major capsid protein (MCP) sequence. Reactions included 20 ng DNA · μL^-1^ in 20 μL reactions with TaqMan Universal PCR Master mix (Life Technologies), 300 nmol forward rtMCP primer (5-ACACCACCGCCCAAAA GTAC-3), 900 nmol reverse rtMCP primer (5-CCGTTCATGATGCGGATAATG-3), and 250 nmol of rtMCP- probe (5-FAM-CCTCATCGTTCTGGCCATCAACCAC-TAMRA-3). gBlocks® gene fragments (Integrated DNA Technologies, Inc.) specific to the *ranavirus* major capsid protein (MCP) sequence [52] were included in each 96-well qPCR plate as standards in log_10_ increments (10^0^ to 10^6^ copies) for quantification of viral concentrations in our samples.

### Statistical analyses

To assess Bd prevalence, swabs were categorized as Bd-positive when Z_e_>0 and as Bd-negative when Z_e_ = 0. For Rv prevalence, samples were considered Rv-positive with qPCR cycles of C(t) < 34; a standard known to exclude false-positives. Sample sizes varied across analyses because some frogs were successfully assayed for only one of either parasite (sample sizes reported for each analysis). We calculated infection prevalence for Bd and Rv by dividing the number of infected frogs by the total number of assayed frogs. We used the R package binom to compute binomial 95% credible intervals intervals using Bayesian inference using Jeffrey’s non-informative priors. To test if parasite abundance within individuals (zeros included; [53]) is associated with the probability of co-infection, we used logistic regression with parasite abundance as a predictor and either Bd or Rv infection status (i.e. infected or not) as a response variable. We then tested for facilitation among these parasites by correlation analysis of Bd and Rv infection intensity (zeros excluded; [53]). Note that we parsed the samples into three distinct populations for these analyses. First, we analyzed the captive *T*. *marmoratus* separately from wild frogs because this population, held in high densities, was unique in their high parasite exposure and co-infection status (*χ*^2^ = 20.5, *P <* 0.001). Second, we compared all wild frogs using logistic regression with frog taxa and parasite abundance as predictors of the probability of co-infection. Last, we tested for elevation effects on parasite prevalence, intensity, and abundance by focusing only on the genus *Pristimantis;* the most widely distributed taxa for which we had sufficient sample size. Here we used logistic regression with elevation class and parasite abundance as predictors of the probability of co-infection.

## Results

**Fig 1:**
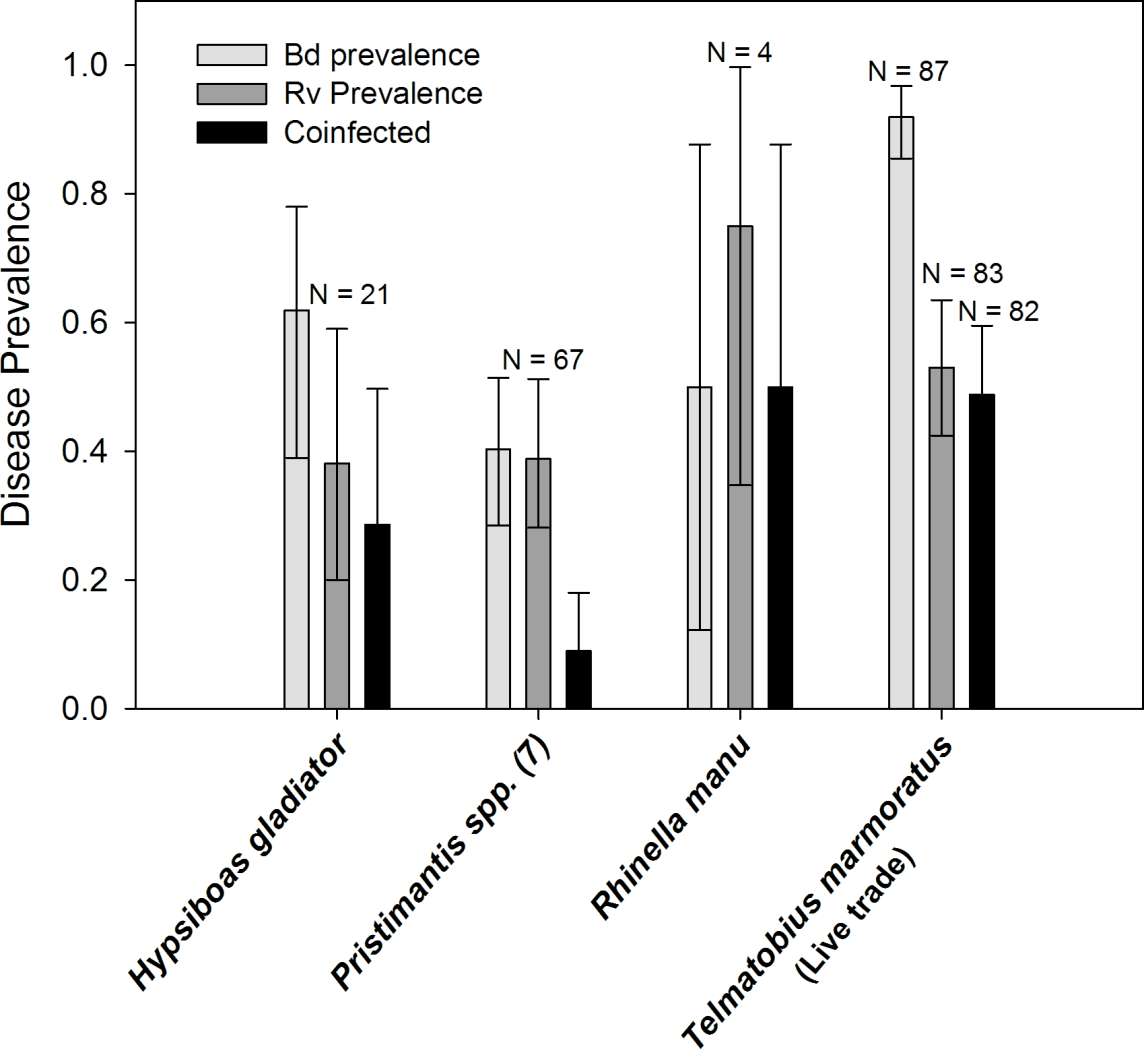
Prevalence of infection by the emerging pathogens *Batrachochtrytium dendrobatidis* (Bd) and *Ranavirus* (Rv) in frogs sampled during 2012 (live trade only) and 2013 (wild and live trade frogs) in Peru. While *Telmatobius* were sampled from live trade sources, the other species were wild caught. Error bars are 95% Bayesian credible intervals using Jeffreys prior.

**Fig 2:**
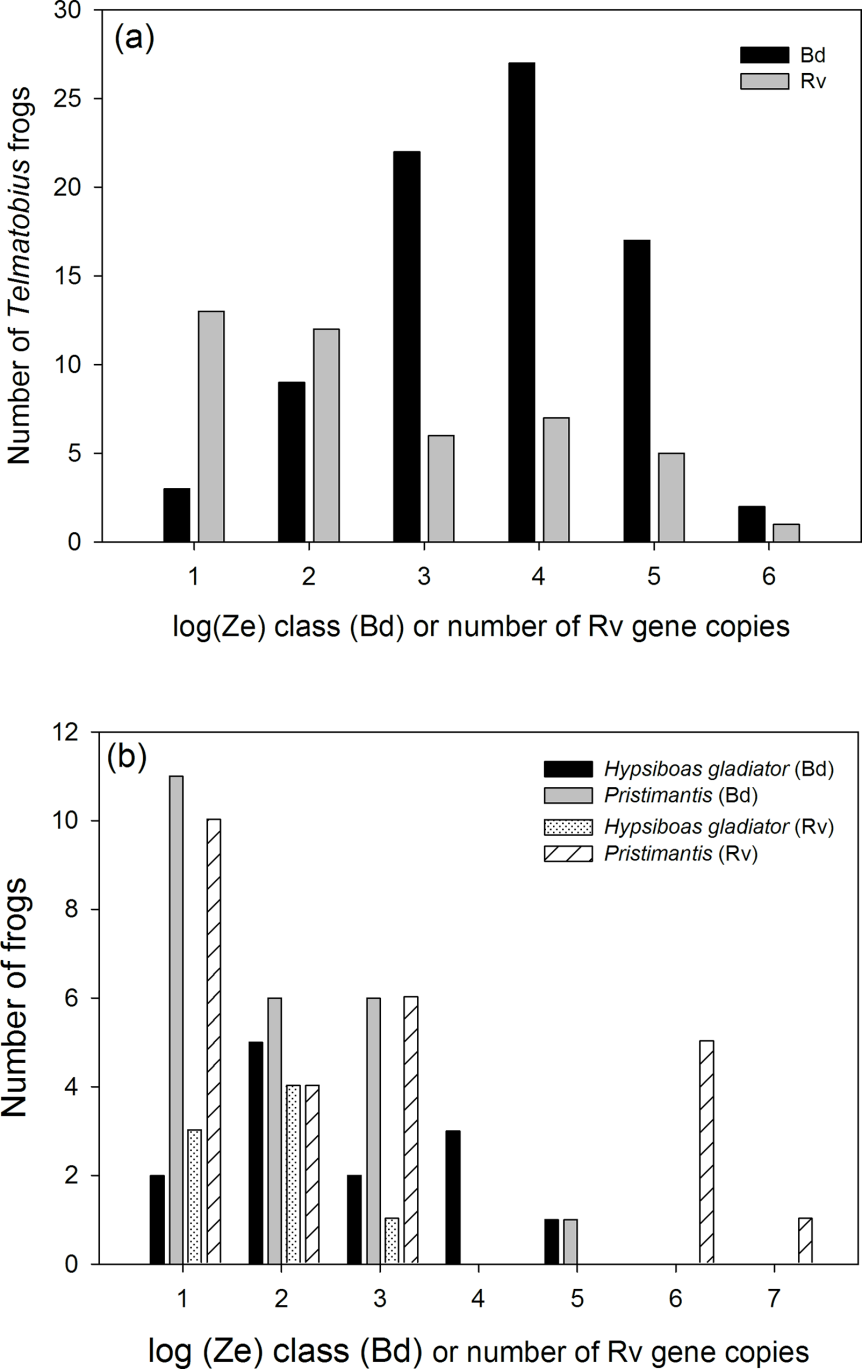
Infection loads in captive *Telmatobius* for both Bd and Rv (a); and in wild stream breeding *Hypsiboas gladiator* and in 7 species of terrestrial, direct-developing *Pristimantis* frogs (b).

*Telmatobius marmoratus* sampled from the live trade in Cusco were heavily infected by Bd (Table 1; prevalence = 91.9%, Credible Interval = 85.5–96.8%, n = 87), Rv (prevalence = 53.0%, CI = 42.4–63.5%, n = 83), and co-infected (Fig 1; prevalence = 48.8%, CI = 38.1–59.5%, n = 82). Bd infection intensity ranged from 10^1^ to 10^6^ zoospore equivalents (median = 10^3.1^ Ze, n = 80). 58% of individuals had Bd infection intensity values above 1,000 zoospore equivalents and 24% were above the 10,000 zoospore threshold that is often associated with a high likelihood of mortality [6, 54] (Fig 2A). Rv infection intensity ranged from 10^1^ to 10^6^ virion gene copy equivalents (median = 10^2.7^), and 43% of individuals had Rv viral loads above 10^3^ viral copies (Fig 2A). There was no association between Bd and Rv infection intensity (excluding zeros for uninfected) among co-infected *Telmatobius* frogs (*r* = 0.22, *P =* 0.17, n = 39). The abundance of either parasite within individuals (including zeros for uninfected) was not associated with the probability of infection by the other (Bd:*χ*^2^ = 0.39, *P =* 0.53; Rv:*χ*^2^ = 2.0, *P =* 0.16).

**Table 1:** *Batrachochytrium dendrobatidis* and *Ranavirus* infection prevalence in ten species of frogs from Peru. Total sampled is the number of frogs examined, but does not always represent the number of samples for each assay (see text for details). 95% Bayesian credible intervals using Jeffrey’s priors.

Species	Source	Developmental Mode/ Habitat	Bd Infected	Prevalence & CI	Rv Infected	Prevalence & CI	Co-infected	Prevalence & CI	Total Sampled
*Hypsiboas gladiator*	Wild	Aquatic larvae/ Lotic	13	61.9% (39.0–78.0%)	8	38.0% (20–59%)	6	28.6% (12.9–49.7%)	21
*Pristimantis danae*	Wild	Direct/ Terrestrial	1	–	0	–	0	–	2
*Pristimantis fenestratus*	Wild	Direct/ Terrestrial	1	–	0	–	0	–	1
*Pristimantis lindae*	Wild	Direct/ Terrestrial	1	33.3% (1.0–77.1%)	1	33.3% (1.0–77.1%)	0	0% (0–44.4%)	3
*Pristimantis pharangobates*	Wild	Direct/ Terrestrial	1	12.5% (0.1–39.7%)	3	37.5% (10.4–68.6%)	0	0% (0–20.7%)	8
*Pristimantis platydactylus*	Wild	Direct/ Terrestrial	11	34.4% (19.3–51.0%)	13	41.9% (25.6–59.0%)	3	9.7% (1.8–21.6%)	32
*Pristimantis reichlei*	Wild	Direct/ Terrestrial	4	66.7% (32.0–94.6%)	0	0% (0–26.4%)	0	0% (0–26.4%)	6
*Pristimantis* sp.	Wild	Direct/ Terrestrial	1	–	0	–	0	–	1
*Pristimantis toftae*	Wild	Direct/ Terrestrial	7	46.7% (23.6–70.3%)	8	53.3% (29.7–76.4%)	3	20.0% (4.3–41.5%)	15
*Rhinella manu*	Wild	Presumed direct/ Terrestrial	2	50% (12.2–87.7%)	3	75% (34.7–99.7%)	2	50% (12.2–87.7%)	4
*Telmatobius marmoratus*	Live Trade	Aquatic larvae/ Streams and wetlands	80	91.9 (85.5–96.8%) (n = 87)	44	53.0 (42.4–63.5%) (n = 83)	40	48.8 (38.1–59.5%) (n = 82)	88

Among the wild frogs sampled along the eastern slopes of the Andes, there was no difference in infection prevalence between stream/riparian dwelling *Hypsiboas* and terrestrial, direct-developing *Pristimantis* frogs (Fig 1; Bd odds ratio = 0.46, 95% CI = 0.17–1.21, *P =* 0.14; Rv odds ratio = 1.45, 95% CI = 0.5–4.2, *P =* 0.61). *Ranavirus* infected three of the four *Rhinella manu* sampled, while two of these frogs were infected by Bd (Fig 1). In *R*. *manu*, median Bd infection intensity was low 0.5–0.7 Ze, while median Rv infection intensity was 10^4^ viral copies (range = 10^1.6^ to 10^5^). Among the stream-breeding *Hypsiboas gladiator* (n = 21), 65% were infected by Bd, 40% by Rv, and 30% were co-infected; the difference in Bd and Rv prevalence was not significant (Table 1; odds ratio = 2.1, 95% CI = 0.3–15.35, *P =* 0.64). Bd infection intensity for *H*. *gladiator* ranged broadly from 0.5 to 10^5^ zoospore equivalents (Fig 2B; median = 10^1.7^ Ze, n = 13). Rv infection intensity ranged from 10^1^ to 10^3.3^ virion gene copy equivalents (Fig 2B; median = 10^2.1^, n = 13). There was no association between Bd infection intensity and Rv loads among co-infected wild frogs (*r* = -0.35, *P =* 0.22, n = 14). The abundance of Bd within individuals was not associated with the probability of Rv infection (*χ*^2^ = 0.01, *P =* 0.91) or frog taxa (*χ*^2^ = 2.0, *P =* 0.36). Rv abundance within individuals was marginally associated with the probability of infection by Bd (*χ*^2^ = 3.8, *P =* 0.05; log odds estimate for uninfected/infected = 0.25 ± 0.14, *P =* 0.07) but not frog taxa (*χ*^2^ = 2.7, *P =* 0.25).

**Fig 3:**
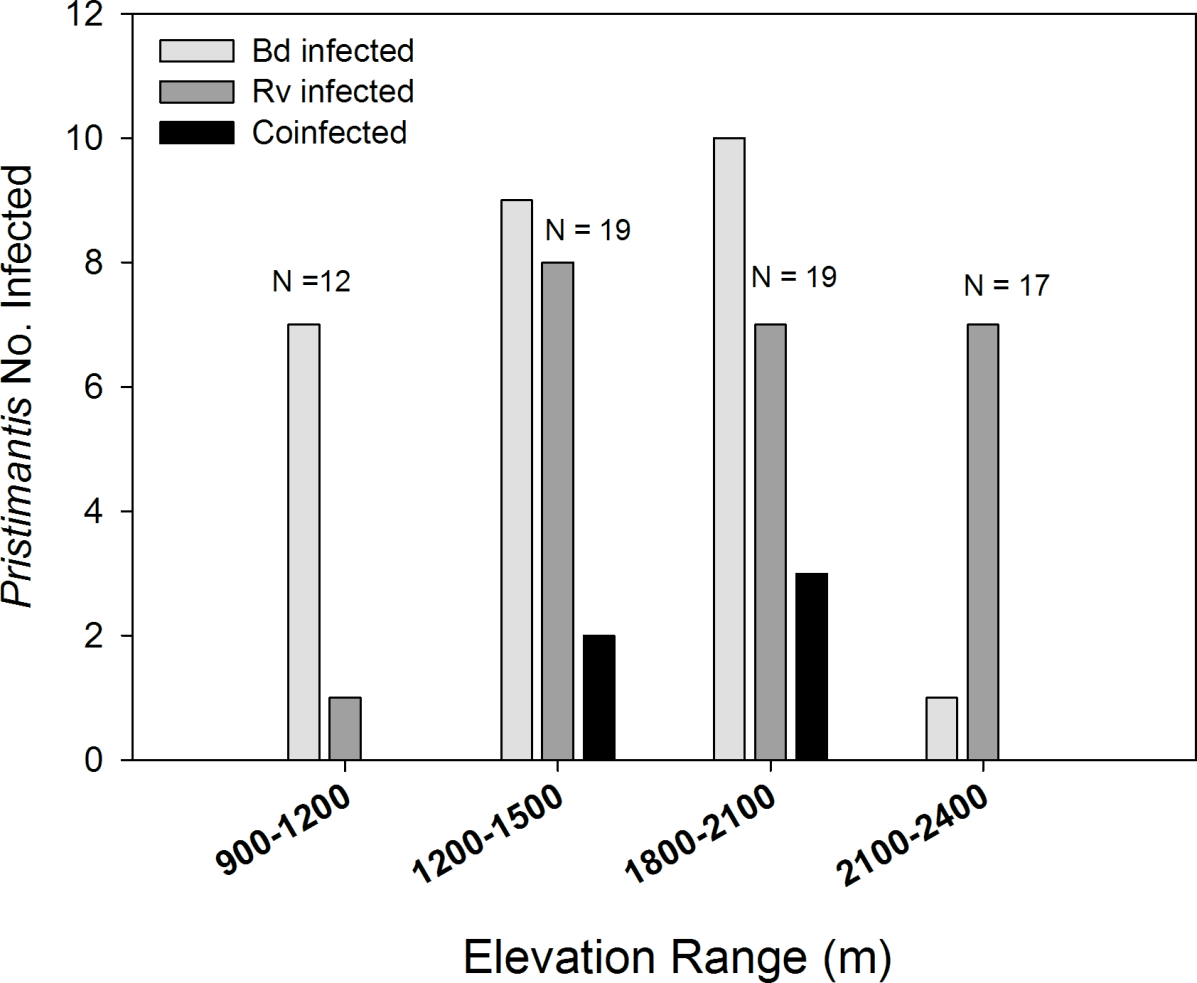
The number of Bd and Rv infections in wild frogs in the genus *Pristimantis* (7 species) varied across an elevation range on the eastern slopes of the Andes (N = total sample size for each elevation range).

Bd and Rv infection prevalence among seven species of direct-developing *Pristimantis* also varied across an elevation gradient ranging from sub-montane forests at 900 meters to cloud forests at 2400 meters (Fig 3). Bd prevalence varied across this gradient (*χ*^2^ = 13.9, *P =* 0.003), driven by a sharp decline in the number of infections above 2100 meters. Rv infections were common across all elevations (*χ*^2^ = 5.4, *P =* 0.15), but lowest below 1200 meters. Co-infection was only present in animals in the cloud forest from 1200 to 2100 meters. Among these seven *Pristimantis* species 39.4% were infected by Bd (Table 1; CI = 28.5–51.4%, n = 68), 38.2% were infected by Rv (CI = 28.1–51.2%, n = 66), but only 9% were co-infected (CI = 4–18%, n = 6; Fig 1); Bd and Rv prevalence was significantly different among *Pristimantis* spp. (odds ratio = 0.26, 95% CI = 0.08–0.83, *P =* 0.02). Bd infection intensity for *Pristimantis* ranged from 0.1 to 10^4^ zoospore equivalents (Fig 2B; median = 1 Ze, n = 68). Rv infection intensity ranged from 10^1^ to 10^7.2^ viral gene copy equivalents (Fig 2B; median = 10^2.6^, n = 68). There was no association of Bd and Rv infection intensity among the few co-infected frogs. Rv abundance within individuals was marginally associated with the probability of infection by Bd (*χ*^2^ = 4.8, *P =* 0.03; log odds estimate for uninfected/infected = 0.45 ± 0.23, *P =* 0.05), as well as elevation (*χ*^2^ = 11.3, *P =* 0.01). These patterns were driven by the decline in Bd, but not Rv infections above 2100 meters (Fig 3; log odds estimate for uninfected/Bd infected = 2.1 ± 0.82, *P =* 0.01). The abundance of Bd within individuals was not associated with the probability of Rv infection (*χ*^2^ = 0.86, *P =* 0.35) or elevation (*χ*^2^ = 6.6, *P =* 0.09).

## Discussion

This is the first report of co-infection by Bd and Rv in South America, and the first report of *ranavirus* infections among amphibians in Peru. These findings raise several crucial conservation concerns for amphibians in the Andes, which is among the most species rich regions for amphibians on Earth [55]. While many factors are threatening amphibians both in Peru and globally, emerging diseases such as Bd induced chytridiomycosis are a primary factor contributing to extirpations and even extinctions among amphibians. However, the extent to which interactions among multiple emerging diseases may be contributing to population declines is unknown.

Several studies have reported co-ocurrence of Bd and Rv in a number of aquatic communities in North America [15, 16], and more recently Whitfield and Kerby [13] found co-infection in several species of frogs in Costa Rica. While Bd induced chytridiomycosis is undoubtedly a primary driver of recent extirpations and extinctions of amphibians, especially in Central America, the growing reports of Bd and Rv co-infection raise questions regarding the distribution and prevalence of Rv and these co-infections (in addition to other parasites like *Ribeiroia* macroparasites), and to what extent concurrent parasite infections contribute to epizootics [15]. We hypothesized that infection by either parasite could facilitate subsequent infection by the other via processes such as immunosuppression, host resource depletion, and/or tissue disruption. However, our analysis of correlations among Bd and Rv infection intensity, as well as the effects of parasite abundance on the likelihood of co-infection did not suggest such facilitation between Bd and Rv in adult frogs. Indeed, the abundance of Rv in wild frogs was associated with a marginally reduced probability of Bd infection, but this was driven by contrasting ecological patterns of Bd and Rv prevalence in *Pristimantis* frogs at their elevation extremes (Fig 3). We do not believe, however, that our results are a definitive test of the facilitation hypothesis because both parasites primarily infect larval amphibians and induce the greatest disease and mortality in metamorphic and juvenile frogs; our data is all for adult frogs [32]. Given this, we expect any facilitation to occur in larvae during initial exposure to these parasites and primary infections. Experimental infection trials in a factorial design that accounts for developmental stages are required to truly test for facilitation of co-infection.

Our results do suggest that infected adult frogs could serve as potential reservoirs for both Rv and Bd. What is more, adult frogs may be an important source for epizootic outbreaks among larval amphibian communities if during the reproductive season they shed these parasites into breeding ponds and streams [56]. Reproducing frogs may be induced to shed parasites if they are immunosuppressed during the breeding season, and quiescent infections thus become active. Breeding frogs may be immunosuppressed, because the reproduction can induce physiological stress [57]. During such periods of breeding or development when larvae or frogs are potentially immunosuppressed is when facilitation between Bd and Rv is most likely to be apparent. This view of parasite facilitation assumes that (i) such interactions between parasites and/or their apparent effects on their host are temporary or periodic; and (ii) that the state or condition of a host influences community interactions among co-infecting parasites [34, 36, 37, 58]. If these assumptions are true, then the detection of parasite interactions and their effects on hosts are similar to the detection of life history trade-offs, which are only apparent when animals are physiologically stressed [59, 60]. In the context of our stated hypotheses, correlations of infection intensity between co-infecting parasites, and parasite abundance effects on the likelihood of co-infection would only be apparent during life states in animals when they are most vulnerable due to factors like physiological stress or shifts in critical developmental windows [32]. Given this, we suggest that future tests of co-infection in animals take into account the life stage/state and condition of hosts.

Wild amphibians, and most taxa for that matter, are rarely assayed for multiple infections, especially during critical periods of infection or epizootic events. While chytridiomycosis is likely the ultimate causative agent of mortality in recent amphibian declines, it is possible that susceptibility to Bd infection is shaped by prior or concurrent infections by parasites such as Rv. In addition, Bd infections may be contributing to the spread and invasion of other emerging pathogens such as Rv, if these parasites facilitate multiple infections via immunosuppression and host resource depletion [13]. Alternatively, it is possible that Rv is endemic to Peru and does not strongly influence amphibians in this region. However, without greater surveillance and data it is impossible to determine the threat posed by Rv and other potential co-infecting parasites. Therefore, future surveys and amphibian conservation programs focused on Bd should also test for the presence of Rv and monitor for signs of secondary infections. In addition, broad surveys of Rv are warranted to determine if this parasite is a threat to many already endangered amphibian species.

While Rv epizootics can be devastating, causing greater than 90% mortality among larvae in an affected pond, these outbreaks are often rapid and sporadic, which make them difficult to detect. In Peru, however, a recent report of diseased adult *T*. *marmoratus* frogs near Cusco described animals exhibiting signs (lesions, edema) consistent with *ranavirus* infection (A. Ttito, *pers*. *comm*.). *Ranavirus* induced disease in adult amphibians is alarming because this could suggest a highly virulent strain is present in Peru; most Rv induced mortality is in metamorphic larvae [32]. Reports of *ranavirus* infections and associated epizootics in Central America also could suggest that this disease is widespread and of potential conservation concern [13, 61]. In addition, *ranaviruses* are not host-specific so a single virulent strain can infect fish, reptiles and amphibians [23, 26–29], which suggests that this pathogen can threaten entire wetland communities; as was recently described in *ranavirus* epizootics in Spain [10].

While *ranavirus* outbreaks occur sporadically [62, 63], the factors driving the cryptic pattern of disease outbreaks are likely influenced by variation in susceptibility among species [30] and life stages, with larvae nearing metamorphosis being most susceptible possibly due to the stress associated with metamorphic tissue remodeling [32, 64, 65]. *Ranavirus* emergence and disease outbreaks may also be linked to environmental change like altered habitats, changes in water chemistry, pollution and climate change [33]; as well as potentially the stress imposed by infection with other diseases like Bd. Clearly, to understand the extent which Rv is distributed in Peru and South America, its prevalence, and any threat it poses to amphibian conservation in conjunction with other parasites, we need more extensive and thorough surveys and assays testing for animal health [66]. Last, genome sequencing of Rv strains present in Peru and other regions of the Americas could shed light on variance in distribution of differing strains, and potentially the means of spread of these emergent diseases [10, 67–69].

Rv and Bd are water-borne parasites that are often associated with infections in the aquatic, larval life-stage of most amphibians, and which impose the highest mortality during the metamorphic and post-metamorphic life-stage of aquatic amphibians. Our study documents for the first time Rv and Bd co-infection in *Pristimantis*, which are largely terrestrial frogs that lay their eggs in leaf litter and exhibit direct-development (i.e. small froglets hatch directly from eggs with no free-swimming larval stage) and that comprise a sizable component of amphibian diversity in the Andes [70, 71]. Because these frogs and their larvae interact little with ponds or streams, our finding of high infection rates in *Pristimantis* frogs is rather surprising. We can only speculate about the source(s) and mode of Bd and Rv transmission, but a likely source is environmental. Bd spores and free Rv virions may be present in the moist leaf litter, and spread to these habitats by infected individuals [17, 72]. Another source of exposure could be when these frogs forage in or along riparian habitats that harbor these parasites [17]. Last, shedding of virus from reproducing frogs may also be a source of transmission [56], if infected parental frogs shed Rv or Bd onto the hatchlings, and leaf litter during egg laying and fertilization or if they exhibit parental care. Further research is needed to characterize these potential patterns of Rv and Bd presence in terrestrial habitats and their transmission dynamics.

An important means of potential Rv and Bd spread into these wild communities, with critical conservation implications, is infections in captive *Telmatobius* frogs, that are wild-caught and harvested for human consumption [18–21]. Captive animals are kept in high densities in communal tubs that favor the transmission of diseases, and dead animals as well as their water are likely discarded without concern for the release of diseases to the environment [21]. Thus, captive animals might become spreaders of pathogens that can spill over to wild populations. Indeed, disease spillover from confined market to wild amphibian populations is a probable route for the spread of *ranaviruses* in the United States [22, 69]. Clearly, the patterns of Rv and Bd co-infection we have characterized in captive and wild populations in Peru warrant concern and greater testing. To address this likely threat that the live-trade poses for disease spread, outreach and education are greatly needed to inform the sellers and consumers of the risks of live-trade frogs for disease spread to wildlife.

In fact, seventy-four percent of *Telmatobius* species are threatened, and a quarter of these threatened species are in the category of Critically Endangered in the IUCN Red List [46]. The genus *Telmatobius* is endemic to the Andes, where it occurs from Ecuador to Argentina and Chile, with the largest center of diversification in Peru and Bolivia. Chytridiomycosis has been associated with population declines of *Telmatobius* throughout the Andes [5, 17, 20, 73–77]. For example, the three *Telmatobius* species known from Ecuador were extirpated in the 1990s and are now thought to be extinct [75]. The last individuals of *Telmatobius* found in Ecuador showed symptoms of chytridiomycosis [75]. Moreover, population declines of *T*. *marmoratus*, *T*. *mendelsoni* and *T*. *timens* in Peru [5, 17, 20, 76] and of three species of *Telmatobius* in Argentina [73] have been associated with outbreaks of Bd, and high prevalence of Bd infection has been reported from high-elevation populations of *T*. *jelskii* in central Peru [48] and *T*. *gigas* in Bolivia [77]. While Bd is thus known to threaten these [21] and other vulnerable frogs in Peru [17], the extent to which Rv and co-infection have or are contributing to these declines is unknown. Because amphibians globally are the most threatened group of vertebrates [46], we believe that these co-infection patterns are of great concern and that future research should aim to detect the prevalence of Rv, Bd, and other parasites, as well as test their interacting roles in driving threatened populations to extinction. With concerted efforts, and greatly increased data regarding any such parasite interactions, and the role of humans in spreading these pathogens, we can increase our capacity to contain and mitigate the emergence of these and other wildlife diseases.

## Supporting Information

S1 Fig Sampling locations for wild caught frogs in the Kosnipata valley, near Manu National Park, Cusco, Peru. (KML)
